# PD68 A Cross-Cultural Validation Study Of The German And English Versions Of The ICEpop CAPability measure for Adults (ICECAP-A)

**DOI:** 10.1017/S0266462324003258

**Published:** 2025-01-07

**Authors:** Jasper Ubels, Michael Schlander

## Abstract

**Introduction:**

Proponents of the capability approach argue that the effect of health technologies should be measured in terms of capabilities, that is, the freedom to live as desired. The ICECAP-A, initially developed in the UK, has been used internationally to measure capability wellbeing. This study examined whether participants from Australia, Canada, Germany, the UK, and the USA similarly interpret and respond to the ICECAP-A.

**Methods:**

A multigroup confirmatory factor analysis was conducted. Four types of measurement invariance were tested: configural invariance, metric invariance, scalar invariance, and residual invariance. Measurement invariance was assessed by studying the comparative fit index (CFI) and the root mean square error of approximation (RMSEA) and standardized root mean squared residual (SRMR) fit indices. For this study, data from the multi-instrument comparison database were used to compare response patterns of participants from Australia (n=1,430), Canada (n=1,330), Germany (n=1,269), the UK (n=1,356), and the USA (n=1,460).

**Results:**

The configural invariant model showed adequate fit (CFI 0.992, RMSEA 0.076, SRMR 0.016), and metric invariance was established (change in variables: CFI -0.002, RMSEA -0.014, SRMR 0.015). Scalar invariance (and consequently residual invariance) was not established (change in variables: CFI -0.036, RMSEA 0.046, SRMR 0.018). Post-hoc analysis indicated that full measurement invariance could be established by excluding the German sample, with improved fit index values for configural invariance (CFI 0.994, RMSEA 0.069, SRMR 0.015), metric invariance (change in variables: CFI-0.000, RMSEA -0.020, SRMR 0.006), scalar invariance (change in variables: CFI -0.007, RMSEA 0.011, SRMR 0.006), and residual invariance (change in variables: CFI -0.002, RMSEA 0.009, RMR 0.006).

**Conclusions:**

Response patterns to the German and English versions of the ICECAP-A differed. Caution should be exercised when using these two versions in the same study. Further research is required to determine whether these differences are due to linguistic variations from translation, or whether they indicate fundamental differences in participant understanding and responses to the different versions of the ICECAP-A.

